# Application of mNGS in traceability investigation of foodborne disease outbreaks caused by *Salmonella* Litchfield

**DOI:** 10.3389/fmicb.2026.1870501

**Published:** 2026-08-04

**Authors:** Huarong Hong, Yan Zeng, Zhinan Guo, Shanfeng Yu, Lifang Chen, Liwen Lan, Kunming Wang, Xiaoqin Xu, Yufeng Qiu, Shenggen Wu, Zhifang Zhang

**Affiliations:** 1 Xiamen Center for Disease Control and Prevention, Xiamen, Fujian, China; 2 Xiamen Siming District Center for Disease Control and Prevention, Xiamen, Fujian, China; 3 Xiamen Huli District Center for Disease Control and Prevention, Xiamen, Fujian, China; 4 Fujian Center for Disease Control and Prevention, Fujian Provincial Key Laboratory of Zoonotic Diseases, Fuzhou, Fujian, China

**Keywords:** cold-chain food, metagenomic next generation sequencing(mNGS), multidrug-resistant strains (MDR), *Salmonella* Litchfield, T3SS, tet(A)

## Abstract

**Background:**

*Salmonella* is one of the most common pathogens responsible for foodborne outbreaks, posing a serious threat to public health. However, when conventional culture methods fail to isolate the pathogen from food, identifying the contamination source becomes challenging. Here, we report an investigation of a foodborne outbreak caused by *Salmonella* Litchfield that occurred in Xiamen,China on Sep 22, 2025. Isolation of the pathogen was successful only from clinical specimens, whereas all food and environmental samples tested negative.

**Methods:**

Clinical, food, and environmental surface swab samples were collected. The clinical samples were screened using the 14-plex PCR assay for rapid pathogen detection. Metagenomic sequencing (mNGS) was performed on all samples in parallel. Conventional bacterial culture was also conducted, and the obtained isolates were subjected to whole-genome sequencing (WGS). A SNP-based phylogenetic tree was constructed using WGS data from clinical isolates and reference strains from different geographical regions.

**Results:**

This foodborne outbreak was caused by *Salmonella* Litchfield (sequence type ST124),which was recovered from anal swabs of the six patients, including the chef. Phylogenetic analysis showed that the five patient isolates formed a distinct outbreak clone, whereas the chef's isolate belonged to a separate sublineage. No SNPs differed between the chef and four of the patients; however, the one-SNP difference observed in one patient isolate represented a microevolutionary event during transmission. The chef's isolate was closely related to a strain isolated in Hangzhou 5 years previously, with a 15-SNP difference between them. No pathogens were isolated from any food samples. cgMLSTFinder detected 2,768-2,772 core genes, with completeness >99.71%, and an average GC content of 52.25%. mNGS analysis identified high abundances of *Salmonella* in food samples at the genus level. Among the virulence genes detected, T3SS2 and T3SS, components of canonical virulence systems in *Salmonella*, were present at high abundance. Additionally, *floR* and *tet (A)* were highly abundant in food samples.

**Conclusions:**

Our integrated approach combining culture, WGS, and mNGS proved effective for rapid outbreak traceability, suggesting that the outbreak most likely originated from a contaminated food source associated with cross-regional dissemination, although the specific vehicle and transmission route remain to be determined.

## Introduction

*Salmonella* is a foodborne pathogen primarily found in the intestinal tracts of humans and animals. It is transmitted through contaminated food or water, typically associated with the consumption of undercooked or unprocessed foods. As a common cause of gastroenteritis, it leads to approximately 9,380,000 infections and 155,000 deaths annually (Dos Santos et al., 2019). The *Salmonella* genome comprises two species: *Salmonella enterica* and *Salmonella bongori*. Among these, *Salmonella enterica* encompasses six subspecies and is further classified into over 3,000 serovars (Worley, 2025). The host range and ability to infect specific hosts vary considerably among these serovars (Grimont and Weill, 2007). Most human diseases are caused by serovars belonging to the subspecies *Salmonella enterica* subsp. *Enterica* (Hagedoorn et al., 2023). Given the differences in characteristics and behavior among different serovars of this pathogen, identifying *Salmonella* to the serovar level is crucial for epidemiological investigations (Lu et al., 2025).

Among the *Salmonella* serovars that cause foodborne disease outbreaks in China, *Salmonella* Enteritidis and *Salmonella* Typhimurium are the two most common and dominant. *Salmonella* Litchfield is a serovar of *Salmonella enterica* subspecies *enterica* belonging to the non-typhoidal *Salmonella* (NTS) group (Ballal et al., 2016). It has been occasionally isolated from *Trachemys scripta elegans* (red-eared slider turtle) (Kuroki et al., 2019) and associated with outbreaks in Japan (Okabe et al., 2021), Australia (Gibbs et al., 2009), Spain (Lafuente et al., 2013), and other countries. In China, food poisoning incidents caused by consuming cold dishes have been reported in Hunan (Duan et al., 2010)and Nanjing (Liu, 1994), with *Salmonella* Litchfield isolated from suspected food samples. Similarly, in Guangzhou (Zhang et al., 2012), pathogenic bacteria were successfully cultured and isolated from 17 patient stool samples and one kitchen cloth swab during an outbreak investigation. However, there have been few in-depth genomic analyses of this strain regarding its antimicrobial resistance and virulence genes, either domestically or internationally. Whole-genome sequencing (WGS) is now widely adopted in *Salmonella* epidemiology, enabling precise determination of sequence types and molecular detection of resistance-associated genes or point mutations (Ellington et al., 2017; Diep et al., 2019). Metagenomic next-generation sequencing (mNGS) has introduced novel approaches and perspectives for pathogen identification. When sufficient sequence coverage is obtained, it also enables strain typing, antimicrobial-resistance profiling, source tracing, and early-warning prediction of outbreaks (Gu et al., 2021).

In September 2025, a foodborne outbreak caused by S*almonella* Litchfield occurred in Xiamen City in China. By integrating conventional bacteriological methods with WGS and mNGS, we identified a distinct outbreak clone among the five patients. Although the isolate from the chef showed close genomic similarity to this clone, it lacked one virulence factor and occupied a separate phylogenetic position. These findings effectively rule out the chef as the direct source of contamination. Instead, the data are most consistent with a outbreak in which the patients were exposed to a food item that was already contaminated before handling, although the specific vehicle remains unidentified. These observations underscore the importance of cross-regional food supply chain surveillance in foodborne disease prevention, particularly for cold-chain products distributed along China's southeastern coast. This was highlighted by our investigation of a *Salmonella* outbreak in Xiamen, in which the application of WGS and mNGS, combined with conventional epidemiology, unexpectedly uncovered a likely link to cold-chain food products originating from the Yangtze River Delta region.

## Materials and methods

### Epidemiology investigation

Case definition:All individuals who dined at Restaurant A (Anonymized to protect privacy) in Xiamen City between Sep 17 and Sep 22, 2025 (the restaurant began operation on Sep 17 and suspended business on the evening of Sep 22), along with those who tested positive for *Salmonella* using the VITEK 2-compact system combined with conventional culture and serological confirmation, or developed at least one of the following symptoms: diarrhea (≥3 bowel movements with altered consistency within 24 h of onset) or vomiting (≥2 vomiting episodes within 24 h of onset). Individuals who dined at the restaurant and restaurant staff were identified according to the case definition, while related cases were also searched through the Foodborne Disease Surveillance and Reporting System. Case Investigation: Field investigators from the Center for Disease Control and Prevention (CDC) conducted investigations on the identified cases using a standardized questionnaire to collect information, according to Technical Guidelines for the Epidemiological Investigation of Food Safety Incidents (2012 Edition), including basic personal details (name, contact information, date of birth, etc.), demographic data (age, sex, occupation, etc.), time of symptom onset, clinical manifestations, diagnosis and treatment history, as well as dietary exposure within 72 h prior to illness onset. Through site inspection, interviews with personnel, and review of records, the investigation aimed to understand the dining circumstances of those affected in this incident, the handling of suspected food items, cleaning and disinfection of utensils, food sample retention practices, and the health status of food service employees.

### Sample collection

On September 22, 2025, a total of 6 anal swab samples were collected from cases, one of which was from a chef. In addition,4 environmental surface swab samples from food processing areas (interior surfaces of refrigerators, cutting boards, kitchen knives, and scissors), and 5 samples of suspected food or raw ingredients (Eight-Treasure Sauce, Five-Spice Crispy Fish, braised Vegetarian “Chicken” in Spiced Soy Sauce,Soybean Sheets for making vegetarian “chicken,”and Salt-Baked Duck Drumstick) were collected. For each sample type, a single sterile swab was used to swab multiple items of the same category sequentially, and this swab was processed as one sample.

### Initial screening by 14-plex PCR assay

The six clinical samples were screened using a 14-plex PCR assay targeting common foodborne pathogens, including *Salmonella, Staphylococcus aureus, Escherichia coli* O157, *Listeria monocytogenes, Cronobacter sakazakii, Shigella, Yersinia enterocolitica, Vibrio cholerae, Aeromonas hydrophila, Bacillus cereus, Escherichia coli, Vibrio parahaemolyticus, Campylobacter coli*, and *Campylobacter jejuni*. The assay was performed on a Bio-Rad CFX96 Touch Deep Well instrument(Bio-Rad Laboratories, Inc., Hercules, CA, USA) using the 14-foodborne-pathogen detection kit (Jiangsu BioPerfectus Technologies Co., Ltd., China) according to the manufacturer's instructions. Briefly, 0.5 mL of the liquid from each anal swab sample was transferred into a centrifuge tube and vortexed to mix thoroughly. The mixture was then centrifuged at 13,000 rpm for 2 min. The supernatant was completely removed, and 100 μL of nucleic acid extraction solution was added directly to the pellet, followed by thorough mixing. The tube was then placed in a 100 °C water bath for 10 min (with an allowable error of no more than 1 min). After incubation, the sample was centrifuged again at 13,000 rpm for 5 min. Four microliters (4 μL) of the supernatant was used for PCR reaction. The multiplex PCR screening suggested four samples were positive for *V. parahaemolyticus*, but none of these were confirmed by culture. Instead, culture isolated *Salmonella* spp. from six samples in total.

### Isolation and culture

Conventional isolation and culture were also performed on all collected samples. Clinical anal swabs, food samples, and environmental surface swabs were processed according to GB 47894–2016, with appropriate modifications applied only to the clinical specimens. The instrument used was the VITEK 2-compact fully automated microbial identification and susceptibility testing system. Anal swab specimens were mixed in CB transport tubes, and 1 mL of the mixture was inoculated into selenite enrichment broth (SC) using a sterile pipette, followed by incubation at 36.0 °C for 8 h. The enrichment culture was then streaked onto one plate each of the highly selective SS agar, the weakly selective MacConkey agar (MAC), and *Salmonella* chromogenic plate, and incubated at 36.0 °C for 24 h. Suspicious colonies were selected from the plates for identification. Gram staining of the suspicious colonies revealed Gram-negative small bacilli under microscopy. The suspicious colonies were then identified using an automatic microbial identification system. For those biochemically consistent with *Salmonella*, a slide agglutination test was performed using *Salmonella* antiserum. The quality control strain was *Salmonella* Typhi CMCC(B) 50071 (Guangdong Huankai Microbial Technology Co., Ltd.).

### Metagenomic next-generation sequencing and analysis

Anal and food samples as well as environmental surface swabs were shaken with normal saline, subjected to high-speed centrifugation, and stored at −80 °C. Metagenomic sequencing was performed on the Illumina NovaSeq 6,000 platform using a PE150 strategy. A total of 6 Gb of raw data was generated. After preprocessing, clean data were obtained and assembly analysis was performed using the MEGAHIT (https://www.metagenomics.wiki/tools/assembly/megahit) software.

The gene catalog was aligned against the Micro_NR database to obtain species abundance tables at various taxonomic levels. The Micro_NR database is a microbial analysis database derived by extracting microbial sequences of four categories—bacteria, fungi, archaea, and viruses—from the NR() database. Using the DIAMOND software (https://github.com/bbuchfink/diamond/), the unigene sequences are aligned against the bacterial, fungal, archaeal, and viral sequences extracted from the NCBI NR database. We used Krona (https://github.com/marbl/Krona/wiki/) to visualize the species annotation results, showing the relative abundance of species at different taxonomic levels. For functional annotation, abundance analysis of bacterial pathogen virulence factors was conducted based on commonly used functional databases and the gene catalog, utilizing the Virulence Factor Database (VFDB, http://www.mgc.ac.cn/VFs/main.htm). Regarding resistance gene annotation, the Comprehensive Antibiotic Resistance Database (CARD, https://card.mcmaster.ca/analyze/rgi) was used for annotation, yielding the abundance distribution of resistance genes and the taxonomic attribution of these resistance genes.

### Whole genome sequencing and **bioinformatics** analysis

After nucleic acid extraction using a bacterial nucleic acid extraction kit, the strains were sent to Beijing Novogene Technology Co., Ltd. for subsequent library construction and sequencing. The library was constructed through steps including end repair, A-tailing, adapter ligation, fragment size selection, PCR amplification, and purification. Following library quality inspection, paired-end sequencing was performed on the Illumina NovoSeq 6000 sequencing platform with a sequencing depth of 100 × . The raw sequencing reads were subjected to quality control using FastQC (v0.11.9), and the raw data were filtered using fastp (v0.20.0). The sequencing data volume was 1 Gb per strain, with Q20 > 90% and Q30 > 85%. The filtered high-quality reads were then assembled and analyzed using BioNumerics 7.6 software. The assembled sequences were uploaded to the PubMLST (https://pubmlst.org/) database for comparison to obtain Sequence Type (ST) information. Screening for virulence factors in the bacterial genomes was performed based on the VFDB. *Salmonella* pathogenicity islands (SPIs) were identified using the SPIFinder tool available at the Center for Genomic Epidemiology (CGE) website (https://www.genomicepidemiology.org/services/). Acquired antimicrobial resistance genes in the bacterial genomes were identified using the v2.1ResFinder-4.7.2 (http://genepi.food.dtu.dk/resfinder) and CARD databases.

### Phylogenetic analysis

To determine the phylogenetic position of *Salmonella* Litchfield in the global context during this event, 105 representative genomic sequences from the NCBI database (29 from China and 76 from other Countries and regions) were included in the cgSNP analysis. The software snippy (v4.6.0) was used to construct a phylogenetic tree based on cgSNPs, using GCA_040956515.1 as the reference strain.The tree was rooted using the reference sequence GCA_023844575.1. Pairwise SNP distances between strains were calculated using snp-dists (https://github.com/tseemann/snp-dists), and the phylogenetic tree was visualized and annotated using Chiplot (https://www.chiplot.online/).

### Statistical analysis

During the analysis of mNGS data, only data that underwent adapter trimming, low-quality read removal (Q ≤ 5 exceeding 50% of read length), ambiguous base filtering (N >10%), and host read depletion (using Bowtie2) were included for analysis. Due to the high proportion of zero values and the right-skewed distribution observed in the abundance data of ARGs and virulence factors, median and interquartile range (IQR) were used as the primary descriptive statistics. For graphical comparisons, the abundance values were log10-transformed, and boxplots were employed to visualize inter-group differences, effectively illustrating both central tendency and dispersion. The comparison between groups was performed using the Mann-Whitney U test. We performed statistical analyses in Python3.10.5. To account for multiple comparisons, *p*-values were adjusted using the Benjamini-Hochberg false discovery rate (FDR) method. An adjusted *p*-value (*q*-value) < 0.05 was considered statistically significant.

## Results

**Figure 1:**
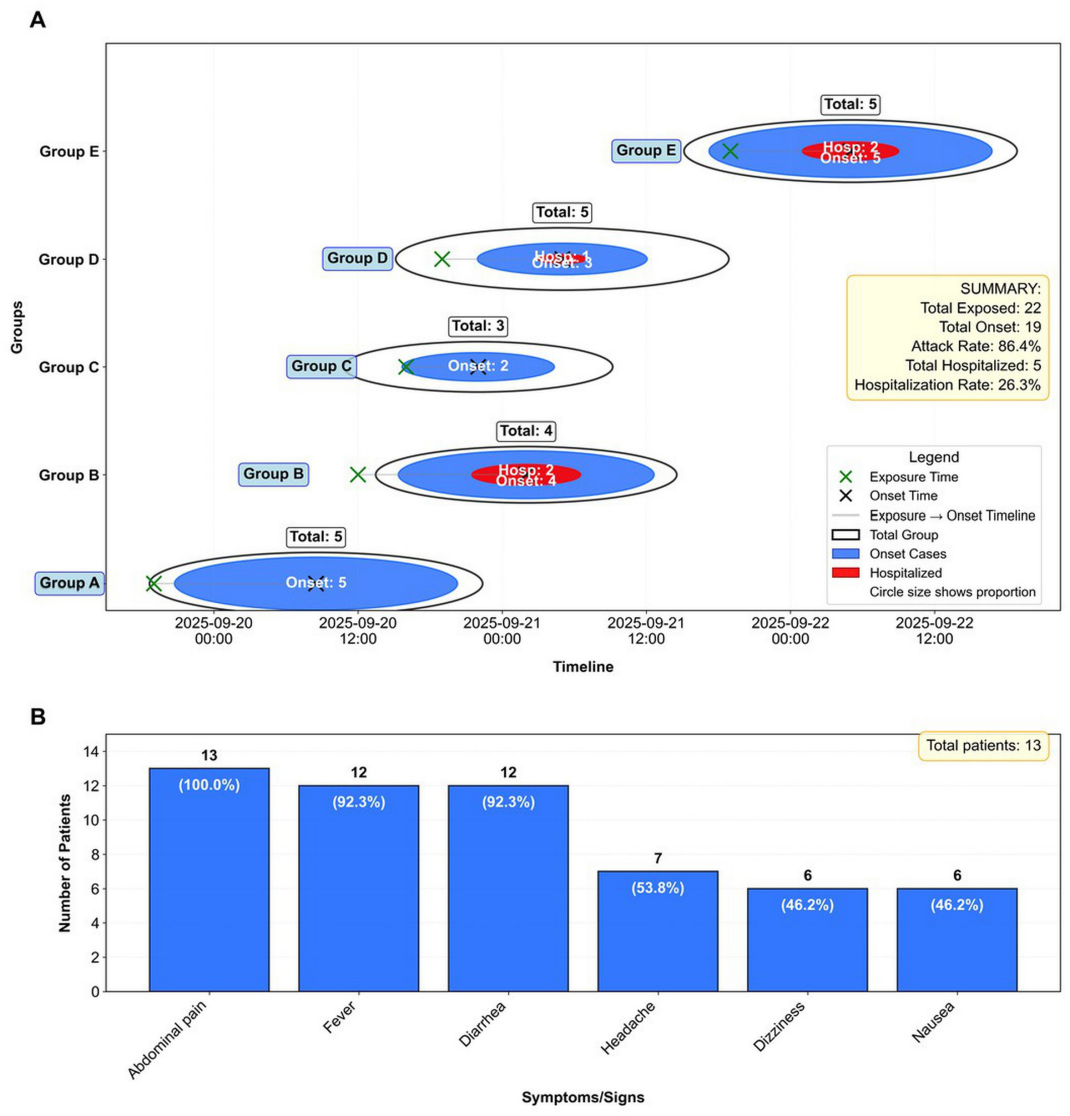
Epidemiological curve and clinical characteristics of cases in a foodborne *Salmonella* Litchfield outbreak. **(A)** Epidemic timeline with cases distribution; (**B)** Clinical symptoms distribution

At about 17:20 p.m. on Sep 22, 2025, SM District CDC of Xiamen received a telephone report that multiple patients, suspected of having dined at the same restaurant at different times, presented with symptoms of acute gastroenteritis, including diarrhea. These patients visited a hospital in Xiamen on Sep 21 and 22, respectively. Upon receiving the report, the municipal CDC and district CDCs immediately dispatched professional personnel to the site to conduct investigation and management. A total of 19 individuals exhibited symptoms, with 5 requiring hospitalization. Among them, 13 met the case definition. The distribution of onset times is detailed in Figure 1A.

## Epidemiological findings

### Demographic and clinical characteristics

Among the 13 cases, 6 were male and 7 were female. Four were minors aged 4–8 years, while the remaining individuals were aged 25–39 years, with a mean age of 26.2 years at onset. The incubation period ranged from 7-26 h, with a median of 19 hours. Most of the affected individuals initially presented with abdominal pain and diarrhea. The main symptoms are detailed in Figure 1B. The diarrhea was predominantly watery, with a frequency of 3–30 episodes per 24 h. Abdominal pain was mainly periumbilical and typically colicky in nature. Vomiting occurred 2–10 times per 24 h. Complete Blood Count of four patients showed increased white blood cell counts, elevated neutrophil counts, decreased lymphocyte counts, and increased monocyte counts.

### Results of on-site hygienic investigation

Item Five-Spice Crispy Fish is a ready-to-eat cooked food, replenished based on remaining stock. It is delivered from Suzhou to the store via SF Express cold chain, stored at room temperature (approximately 25 °C), and served without further processing. The chef reported having consumed some of Five-Spice Crispy Fish during business hours. Raw materials for the other foods were ordered via a delivery platform 1 day in advance, arriving around 8:00–9:00 a.m. on the same day. After delivery, they were kept at room temperature. Processing of these foods took place from approximately 9:30 a.m. to 11:30 a.m. The prepared foods were kept in open stainless-steel containers at room temperature and were available for customers to order. By Sep 22, no leftover foods from the previous 2 days remained at the time of the investigation.

### Results of pathogen isolation and culture

*Salmonella* Litchfield was detected in anal swabs from all six cases, comprising the chef and five patients, with the serum antigenic typing identified as 6,8:1,V:1,2. Among these, *Staphylococcus aureus* was concurrently detected in 1 patient anal swabs. No pathogens were isolated from any food or environmental surface swabs.

### MLST typing, antimicrobial resistance genes, and virulence factor profiles

The complete genome size of the *Salmonella* isolate identified in this study was 4.73 Mb, and its multilocus sequence type (MLST) was ST124. cgMLST analysis indicated that all 6 isolates belonged to the same sequence type (cgST-25671). The core genome allele counts ranged from 2,768 (chef) to 2,772 (other patients). This negligible difference (only four alleles) is well within the acceptable quality threshold for cgMLST analysis. cgMLSTFinder was used to detect core genes. The analysis yielded completeness >99.71%, genome coverage >90%, ANI >98.8%, and an average GC content of 52.25%.Serovars prediction based on the genomic sequence revealed that the strain exhibited characteristics consistent with *Salmonella* Litchfield.

Analysis of virulence factors based on WGS revealed that 6 isolates harbored 42 virulence factors, with the chef isolate carrying 41 (lacking the *shdA* genes) (Supplementary Table 1). This virulence profile encompassed the core virulence systems essential for *Salmonella*: a Type III secretion system mediating invasion of intestinal epithelial cells (*inv,hilA*), high-affinity iron-acquisition virulence factors (*ent,iro*), genes associated with fimbriae and adhesins (*fim,csg,lpf* ), magnesium ion transporters (*mgtB,mgtC*), stress-response regulatory systems (*phoP/phoQ*), among others. These findings indicated that the isolates possessed a complete and typical chromosomally encoded virulence potential characteristics of *Salmonella*, with pathogenicity consistent with the classical features of this serovars. Using the CARD RGI tool and ResFinder 4.7.2, we identified ARGs with sequence similarity and coverage thresholds set above 90%. A total of 37 resistance genes (spanning 17 antibiotic classes) were detected, revealing a complex acquired resistome that included the aminoglycoside resistance genes *aac(6*′*)-Iaa, aph(3*′*)-IIa, aadA1*, β-lactam resistance genes *bla*_OXA − 10_, amphenicol resistance genes *floR, cmlA1*, quinolone resistance genes *qnrS1*, rifamycin resistance genes *arr-2*, tetracycline resistance genes *tet(A)*, and trimethoprim resistance genes *dfrA14*, confirming these strains as multidrug-resistant.

### Phylogenetic reconstruction and source tracing

**Figure 2:**
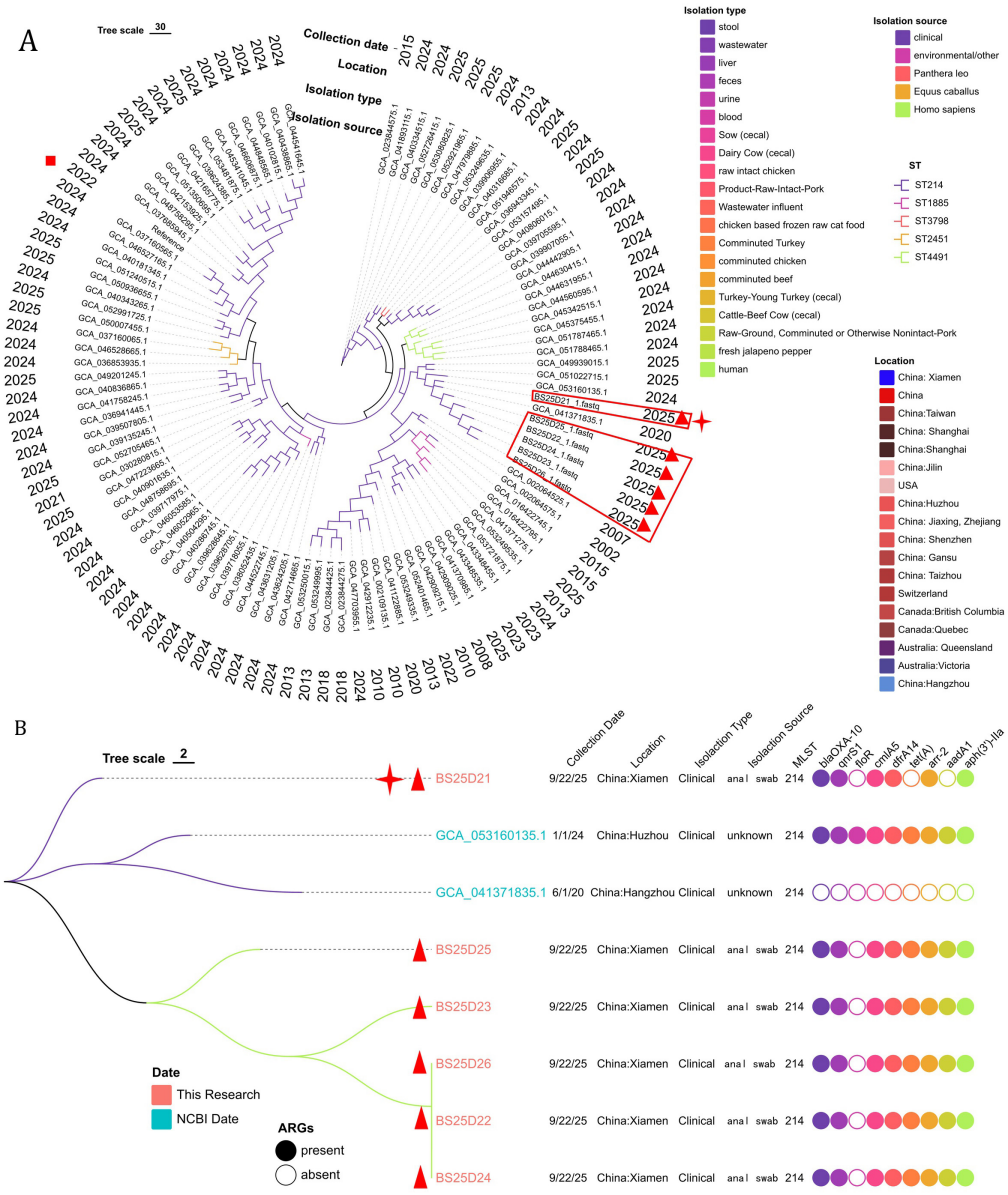
Whole-genome SNP-based phylogenetic analysis of *Salmonella* Litchfield. **(A)** Global phylogenetic tree of 105 strains (76 from NCBI and 29 from the CNCB database), showing the phylogenetic placement of the outbreak strains. 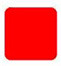 denotes the reference strain, while 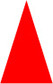 denotes the strain from this outbreak. **(B)** A subtree extracted from (A), including the outbreak strains and the two most closely related strains from Zhejiang Province, 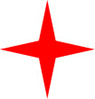 denotes the strain isolated from the chef, while 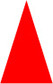 denotes the other outbreak strains.

The phylogenetic tree based on cgSNPs revealed clustering patterns strongly correlated with geographic origin. In this outbreak involving six cases, the five patient isolates formed a distinct outbreak clone, with one isolate (BS25D25) differing by a single SNP as a microevolutionary variant. The chef's isolate, despite high genomic similarity, lacked one virulence factor and clustered separately. Our phylogenetic analysis revealed that the chef's isolate (BS25D21) clustered with a strain from Hangzhou, Zhejiang Province (GCA_041371835.1), with a 15-SNP difference, and both shared a higher-order branch with a Huzhou strain (GCA_053160135.1) that differed by 14 SNPs from the chef's isolate and 9 SNPs from the Hangzhou strain. The five patient isolates formed a distinct outbreak clade (Figures 2A, 2B). In contrast, these outbreak strains differed from the 2 most recent isolates from the United State by more than 70 SNPs, indicating a more distant genetic relationship and a low probability of overseas introduction.

### Abundance and diversity of ARGs and virulence factors in metagenomic data

**Figure 3:**
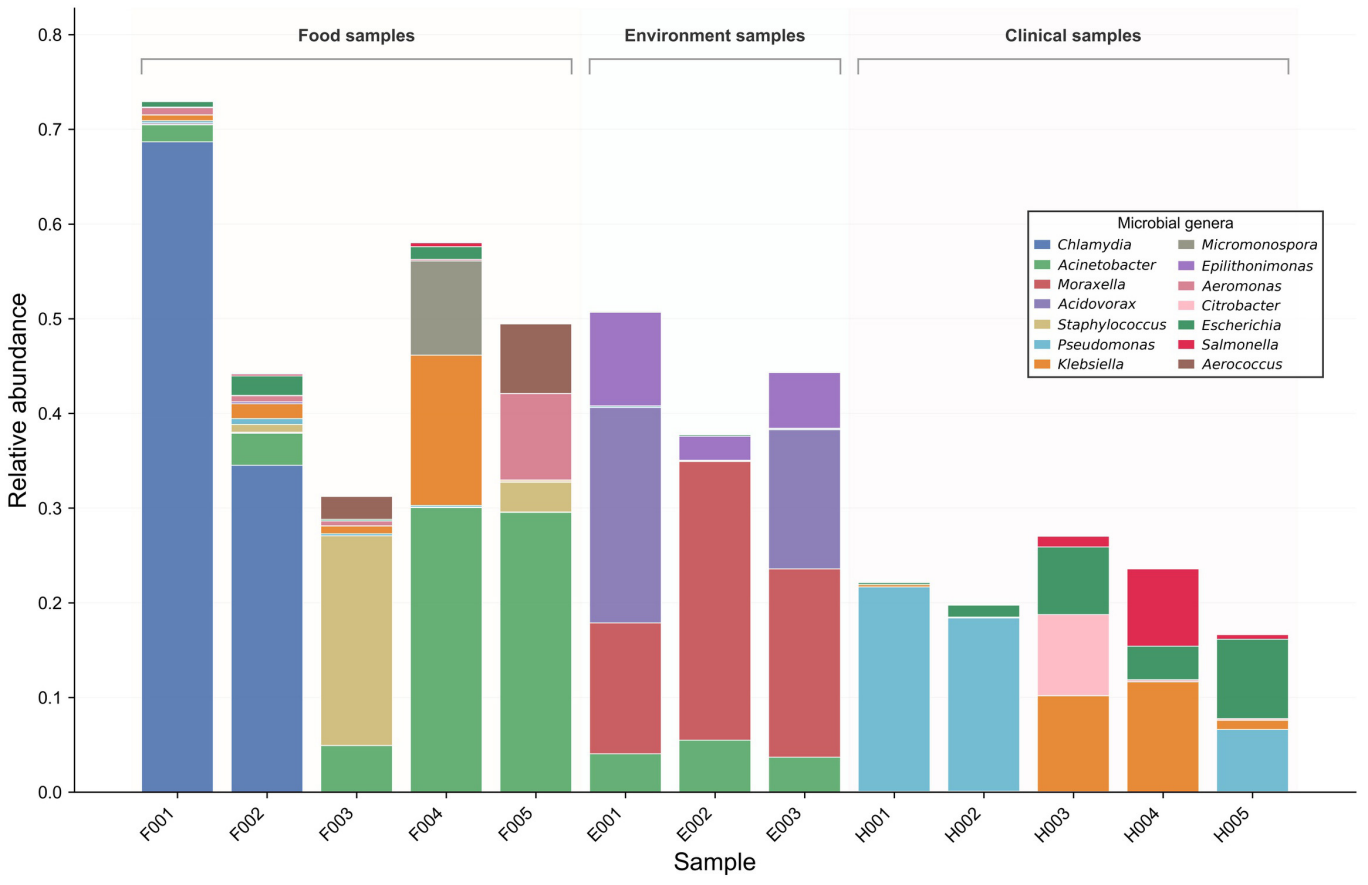
Genus-level taxonomic annotation of 13 metagenomic samples.

**Figure 4:**
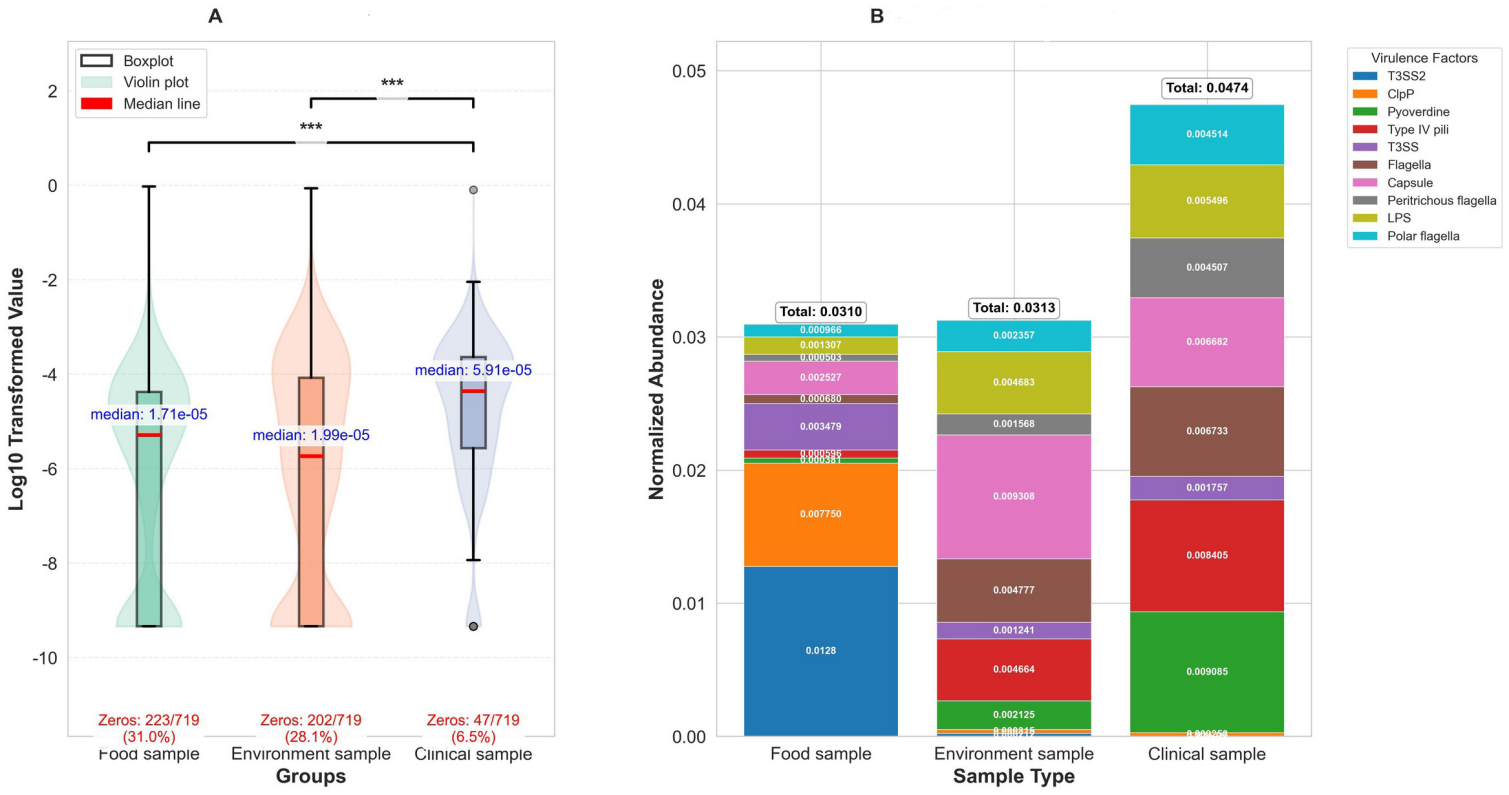
Metagenomic profiling of virulence factors. **(A)** Boxplot and violin plot of all virulence factors in food, environmental, and clinical sample groups (***p < 0.001,**p < 0.01,*p < 0.05,ns *p* ≥ 0.05); **(B)** Stacked bar plot of the top 10 virulence factors across food, environmental, and clinical sample groups.

**Figure 5:**
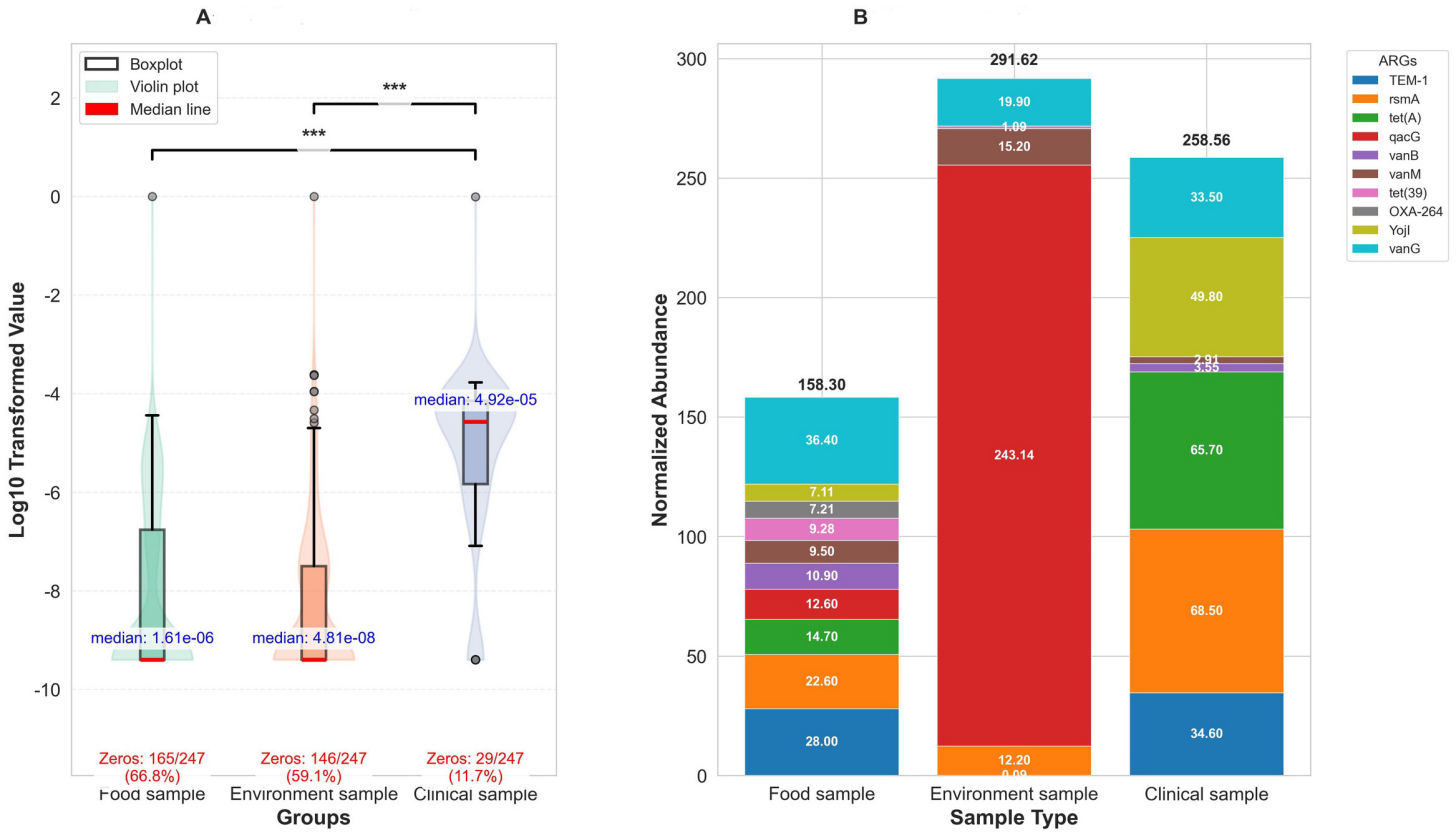
Metagenomic profiling of ARGs. **(A)** Boxplot and violin plot of all ARGS in food, environmental, and clinical sample groups (***p < 0.001,**p < 0.01,*p < 0.05,ns *p* ≥ 0.05); **(B)** Stacked bar plot of the top 10 ARGS across food, environmental, and clinical sample groups.

In this study, mNGS was performed on the submitted samples, with 5 food samples, 3 environmental surface swabs, and 5 patient anal swab samples. Species-level taxonomic annotation revealed that 3 food samples and 4 clinical samples contained high abundances of *Salmonella* at the genus level (Figure 3). Due to the short read length (PE150) and uneven coverage of the mNGS data, reliable *in silico* serotyping could not be performed. In terms of functional gene load, significant differences were observed among the food, environmental, and clinical groups in the overall abundance of virulence factors and ARGs. To further elucidate the shared and unique gene sets across the three groups, a Venn diagram was constructed based on gene presence profiles. The results showed that 5,463 genes were shared among all three groups (Supplementary Figure 1). The Mann-Whitney U test revealed a statistically significant difference in the total number of virulence factors between the food sample group and the clinical sample group (*p* < 0.0001), whereas the difference between the food sample group and the environmental sample group was not significant (*p* = 0.3105) (Figure 4A). Regarding virulence factors T3SS2 was identified at high abundance in the food group (Figure 4B), among which T3SS2 and T3SS are core components of canonical *Salmonella* virulence systems and mediate bacterial invasion and intracellular parasitism. Additionally, several resistance genes overlapping with those found in clinical isolates were detected, such as the *floR* gene. While the difference in the total number of ARGs between the food sample group and the clinical sample group was statistically significant (*p* < 0.0001), the comparison between the food sample group and the environmental sample group showed no significance (*p* = 0.8071) (Figure 5A). In terms of resistance genes, *vanT(G), tem-1, rsmA*,*tet(A)* and *qacG* were the most abundant (Figure 5B).

## Discussion

In recent years, *Salmonella* has emerged as a significant global foodborne pathogen, with frequent outbreaks posing serious threats to public health. Approximately 70%-80% of bacterial foodborne illnesses in China are associated with *Salmonella* infection (Zhang et al., 2018). The incidence rate of foodborne diseases caused by this pathogen is approximately 1,295.59 cases per 100,000 population (Fan et al., 2024). The prevalence and dominant serovars of *Salmonella* in food-producing animals and their products exhibit dynamic evolution, varying significantly across regions and over time. Therefore, it is essential to develop region-specific and temporally adaptive prevention and control strategies (Ferrari et al., 2019; Chen et al., 2024). All 13 cases in this incident had a history of dining at the same catering establishment within 72 h before symptom onset, with no other common dining locations identified. The typical incubation period for *Salmonella* infection ranges from 6 to 24 h, while the observed incubation period in this outbreak was 7–26 h, with a median of 19 hours. The main clinical manifestations included abdominal pain, diarrhea, and fever, which are consistent with the clinical features of *Salmonella* infection. Anal swabs from 5 patients and 1 chef tested positive for *Salmonella*, confirming it as the causative agent of this foodborne outbreak. According to the Technical Guidelines for Epidemiological Investigation of Food Safety Incidents and the Judgment Criteria and Management Principles for *Salmonella* Food Poisoning, the incident was determined to be an *Salmonella* Litchfield infection outbreak. The isolation of *Staphylococcus aureus* from a single patient's anal swab was not considered clinically meaningful, as *S. aureus* food poisoning is mediated by preformed toxins and does not require intestinal colonization or infection.

The six *Salmonella* Litchfield isolates obtained in this study carried nearly identical sets of 42 virulence factors (with one isolate carrying 41), covering key functional modules such as invasion, colonization, intracellular survival, nutrient acquisition, and environmental adaptation. The completeness of SPI-1and SPI-2 endows the strain with a dual-phase–invasion and survival pathogenic capability, enabling both rapid acute gastroenteritis and systemic infection, which closely corresponds to the clinical presentation of the patients. The six clinical isolates shared identical antimicrobial resistance gene profiles, indicating that they are multidrug-resistant (MDR) *Salmonella* Litchfield strains. Notably, the presence of the plasmid-mediated quinolone resistance gene *qnrS1* and the broad-spectrum β-lactamase gene *bla*_OXA − 10_ may lead to reduced susceptibility or resistance to first-line therapeutic agents for invasive *Salmonella* (Siddique et al., 2024).

In this study, clinical samples yielded isolates of *Salmonella* Litchfield, while the food and environmental surface swabs returned negative culture results. This seemingly contradictory phenomenon is common in foodborne disease investigations. Robertson et al. (2019) reported a similar situation during a multi-state outbreak involving *Salmonella* Litchfield: despite strong epidemiological evidence pointing to a hatchery, environmental sampling at the site did not detect the strain. This may be attributed to the relatively low contamination level of this serovars in the environment (compared to the simultaneously occurring *Salmonella* Enteritidis outbreak), falling below the detection limit of conventional culture methods. Additionally, as *Salmonella* Litchfield is not a primary causative serovars for foodborne disease, its environmental adaptability and survival capacity may differ from other more common *Salmonella* serovars, leading to decreased load during food processing or storage and making it difficult to isolate (Pye et al., 2023). The six human-derived *Salmonella* isolates harbored complete virulence islands, conferring the ability for epithelial invasion and intracellular survival. These findings align with the patients' clinical presentations and further support this outbreak. To clarify the transmission chain of this outbreak, we enhanced molecular traceability analysis based on WGS by constructing a SNP phylogenetic tree of clinical isolates. This allowed us to trace the contamination source and assess strain relatedness at the molecular level despite negative food and environmental cultures. Furthermore, we performed mNGS on food, environmental, and clinical samples. Although mNGS is known to have limited sensitivity for detecting pathogens in low-biomass samples, we included it as an exploratory approach to complement culture-based methods and to identify potential pathogens that might otherwise be missed.

SNP genotyping technology is based on whole-genome SNPs for strain typing, offering superior discriminatory power for closely related strains, with high resolution, broad genomic coverage, and high genetic stability. Phylogenetic analysis revealed that the five patient isolates formed a distinct cluster, whereas the chef's isolate occupied a separate branch. Although the chef's isolate showed zero SNP differences from four of the five patient isolates and shared identical antimicrobial resistance gene profiles with all patient isolates, it lacked one virulence factor and was phylogenetically distinct. A single SNP variation was identified in one patient isolate (BS25D25), suggesting a microevolutionary event during transmission and indicating that the isolates belong to the same microevolutionary cluster rather than being strictly clonal. These findings indicate a foodborne outbreak of *Salmonella* Litchfield, but the specific transmission route and source remain undetermined due to the absence of positive food.

Previous outbreak investigations have documented the potential role of chefs in *Salmonella* transmission. For instance, a 2007 *Salmonella* Litchfield outbreak in New Jersey, USA, was traced to multiple infected chefs at a single restaurant, with contamination originating from an ill chef (Centers for Disease Control Prevention (CDC), 2008). Furthermore, recent genomic epidemiology studies have estimated that up to 57.52% of invasive non-typhoidal *Salmonella* infections could be attributed to direct human-to-human transmission (Wang et al., 2025). These findings underscore the importance of strengthening health management and hygiene training for chefs in catering settings. In contrast to these human-to-human transmission scenarios, our phylogenetic and genomic data point to a different epidemiological pattern. In the present outbreak, the chef's isolate lacked one virulence factor and occupied a separate phylogenetic branch from the patient outbreak clone, arguing against the chef as the direct source of infection. Instead, the five patient isolates formed a distinct outbreak clone, suggesting common-source exposure to a contaminated food item.

The tree topology revealed that the chef's isolate (Xiamen, September 2025) clustered with a strain from Hangzhou (Zhejiang Province, June 2020), with a 15 SNPs difference over approximately 5.25 years, consistent with the long-term microevolutionary rate of *Salmonella*. Both isolates shared a higher-order branch with a Huzhou strain (from Zhejiang Province, China, 2024), which differed by 14 SNPs from the Xiamen isolate and 9 SNPs from the Hangzhou isolate. This nested phylogenetic structure indicates the circulation of a persistent *Salmonella* clone in the Hangzhou-Huzhou-Suzhou region of Zhejiang and Jiangsu Provinces over at least 5 years, with subsequent dissemination to Xiamen through cold-chain food products from Suzhou. The chef's isolate, while originating from this same regional clone, lacked one virulence factor and occupied a separate phylogenetic branch from the patient outbreak clone, arguing against the chef as the direct source of this outbreak. Instead, the five patient isolates formed a distinct outbreak clone, consistent with common-source exposure to the contaminated cold-chain food item. The epidemiological, phylogenetic, and genomic evidence collectively support a common-source foodborne outbreak linked to a contaminated food item transported via cold chain from Suzhou (Jiangsu Province) to Xiamen (Fujian Province). Collectively, these findings establish a cross-provincial foodborne transmission chain spanning approximately 800 kilometers from the Taihu Lake region (within the Yangtze River Delta, encompassing Hangzhou, Huzhou, and Suzhou) to Xiamen, highlighting the need for enhanced surveillance of cold-chain food supply chains and persistent *Salmonella* clones in inter-regional food trade.

Similar cases have been documented internationally. In 2022, an outbreak of *Salmonella* Mbandaka was identified in Finland. WGS linked the domestic outbreak strain to a previously reported strain in the United Kingdom, and the source was ultimately traced to pre-cooked frozen chicken meat originating from outside the European Union (Gonzalez-Perez et al., 2025). In the same year, the U.S. Food and Drug Administration (FDA) isolated *Salmonella* Weltevreden from a batch of frozen precooked ready-to-eat shrimp imported from India during routine import sampling. Subsequently, nine cases across four states were confirmed, with clinical isolates differing from the product isolate by only 0-14 SNPs (mean: 3.53 SNPs), indicating a high degree of genetic relatedness (Jenkins et al., 2024). These examples highlight that cold-chain foods have become a significant vehicle for the transboundary transmission of *Salmonella*. Once contamination occurs during production or cold-chain transport, pathogens can rapidly cross national borders through the globalized food supply chain, giving rise to multistate or multinational outbreaks. Notably, in both incidents, the precise linkage between sporadic cases and specific imported food batches was only made possible through WGS. Without robust genomic surveillance networks and effective international data-sharing mechanisms, such cross-border transmission events would likely remain undetected for extended periods. Therefore, strengthening genomic surveillance of foodborne pathogens and promoting data interoperability across countries (Zhang et al., 2026) are of critical importance for the prevention and control of cold-chain-associated salmonellosis.

Virulence factor analysis further revealed that the chef isolate lacked the *shdA* gene, which was present in all other patient isolates. *ShdA* is an autotransporter protein that is largely restricted to *Salmonella* enterica subspecies I and has been shown to contribute to efficient and prolonged fecal shedding in a murine model, without affecting the LD_50_ or the capacity to cause acute lethal infection (Kingsley et al., 2000). Subsequent studies further demonstrated that *ShdA* mediates binding to fibronectin and collagen I via its passenger domain, and is required for long-term intestinal colonization rather than for the initial stages of infection (Kingsley et al., 2004). The recovery of a *shdA*-deleted isolate from the chef who developed acute illness is therefore fully consistent with the proposed role of *ShdA* in intestinal persistence and transmission dynamics, rather than in the induction of acute foodborne disease.

The 14 SNPs between the Huzhou (collected at an unspecified time in 2024) and Xiamen (collected in September 2025) strains, accumulated over approximately 1 year, exceeds the expected *Salmonella* evolutionary rate of 1-3 SNPs per year. This apparent acceleration is likely attributable to: (1) prolonged in-host persistence of the strain in the asymptomatic chef prior to sampling; (2) host-derived selective pressures that may elevate mutation rates during chronic carriage; (3) the unspecified isolation month of the Huzhou strain in 2024, which may extend the actual time interval; and (4) the likely existence of unsampled intermediate strains within a continuously evolving regional clonal population, meaning the observed SNPs may reflect divergence across multiple transmission steps rather than direct evolution from a single sampled ancestor.

The high assembly quality achieved in this study (N50 up to 396,335 bp; Q30 > 97%) supports the authenticity of the observed SNP as genuine biological variation, rather than a sequencing artifact. Consistent with our findings, Rathnayake et al. (Rathnayake et al., 2023) reported that outbreak-related *Salmonella* Enteritidis isolates exhibiting 0-1 SNP differences were unequivocally assigned to the same outbreak cluster, noting that fewer than five SNP differences have been considered acceptable for defining outbreak-related clusters in various investigations. Similarly, Cherchame et al. (2022) demonstrated that core-genome SNP phylogenetic analysis successfully identified the same epidemic clone over a 7 year period. Thus, we conclude that the single SNP difference observed in one patient isolate reflects microevolution within the outbreak clone rather than evidence of non-clonal transmission.

We acknowledge as a limitation our inability to identify the specific contaminated vehicle, owing to the unavailability of food or environmental samples for confirmatory testing. mNGS bypasses the cultivation step and enables direct genomic analysis of the microbial composition in samples. Its detection limit is significantly lower than that of culture-based methods, allowing it to detect nucleic acid signals from pathogens with extremely low loads. In recent years, studies abroad have successfully applied mNGS technology to identify and trace pathogens in foodborne outbreak investigations (Buytaers et al., 2021). To trace the clues of pathogenic bacteria in food, we attempted to perform species annotation on samples using mNGS. At the genus level, *Salmonella* was annotated in 3 food samples and 4 clinical samples. However, the *Salmonella* DNA captured by mNGS in food samples may originate from residual DNA of bacteria that were inactivated during food processing. It is important to acknowledge that mNGS itself cannot distinguish between DNA derived from viable vs. non-viable cells. Therefore, even if only dead bacteria or their DNA remnants, rather than culturable live cells, are detected in food samples, the possibility of these samples serving as a potential contamination source cannot be completely ruled out. A plausible explanation is as follows: if viable *Salmonella* cells (potentially below the detection limit of culture methods due to uneven distribution within the same batch) or sublethally injured bacteria are present in the food, these bacteria may resuscitate and proliferate upon entering the favorable environment of the human intestinal tract, leading to host illness. Consequently, positive mNGS results in food samples strongly suggest that the food has been contaminated with *Salmonella* and point to a clear direction for further traceability investigation. Even when viable bacteria cannot be isolated, this finding provides important molecular clues for identifying the suspected food source. Epidemiological investigation identified a suspected food item sourced from Suzhou, located in the Jiangsu-Zhejiang region. mNGS of this food sample further detected reads taxonomically assigned to the genus *Salmonella*, providing supportive microbiological evidence for the epidemiological link. However, due to limitations in sequencing depth and assembly coverage, a complete *Salmonella* genome could not be reconstructed from the metagenomic data, precluding strain-level confirmation or direct comparison with the clinical isolates. Nevertheless, the convergence of epidemiological tracing, phylogenetic analysis of clinical isolates, and mNGS detection points consistently to a contaminated food item originating from the Jiangsu-Zhejiang region as the most likely source of this outbreak.

Although *Salmonella* Litchfield was not directly isolated and cultured from the food samples, the mNGS analysis of the food revealed relatively high abundances of T3SS and T3SS-2, encoded by SPI-1 and SPI-2, respectively. The pathogenicity of *Salmonella* depends on these two independent Type III Secretion Systems (T3SSs), which deliver virulence factors that facilitate host cell invasion, induce and modulate inflammatory responses, and alter the migration properties of infected phagocytes, among other functions. For *Salmonella*, the major Type III effector SopD is essential for pathogenicity, and *Salmonella* stimulates inflammation through Type III effectors SopA and SopD (Jiang et al., 2004). Beyond the core virulence machinery of *Salmonella*, we also detected other virulence-associated operons. These findings suggest that infection is not an isolated act of a single pathogen but occurs within a complex microbial community context, where horizontal gene transfer and bacterial interactions may influence the progression and outcome of infection. Additionally, the detection of the *tet(A)* gene in the food metagenome provides important traceability implications. The *tet(A)* gene mediates active efflux of tetracycline-class drugs. Tetracycline antibiotics, as a class of broad-spectrum synthetic antimicrobial agents, are approved for use in poultry farming in countries and regions including the United States, China, Brazil, and some European Union member states (McEwen and Collignon, 2018), leading to their widespread presence in animal-derived foods (Xin et al., 2024). The residual excess of antibiotics in meat and other daily foods can enter the human body through the food chain (Muriuki et al., 2001), leading to various adverse effects such as poisoning, bacterial resistance, and weakened immune system function, posing a serious threat to human life and health due to antibiotic contamination (Jansen et al., 2018). The detection of homologous ARGs in the microbial communities of food samples and acquired resistance genes in clinical isolates provides indirect molecular evidence of a link between them. When bacteria carrying *tet(A)*resistance gene are consumed by patients, pathogenic bacteria may colonize the intestine and cause infection. Meanwhile, other resistance gene elements carried by bacteria in food may undergo horizontal transfer into the intestinal flora, ultimately integrating into the genome of pathogenic bacteria, potentially explaining the more complex resistance profiles observed in clinical isolates. Such contamination may originate from contact with carriers or reservoirs of resistant bacteria during food production or processing. Additionally, the resistance gene pool in food samples exhibited greater diversity, including vanT(G),TEM-1 and qacG, none of which were detected in the clinical isolates. This disparity suggests that the infection microenvironment may serve as a niche for commensal bacteria that harbor resistance genes, potentially constituting a reservoir that could contribute to the dissemination of resistance determinants.

## Limitations

In this study, clinical samples yielded isolates of *Salmonella* Litchfield, while the food and environmental surface swabs returned negative culture results. *In silico* serotyping was not attempted for the metagenome samples because the sequencing reads were short (PE150 bp) and the coverage across the *Salmonella* genome was uneven. These factors preclude reliable serotype prediction from the metagenomic data. Additionally, due to the lack of data sharing between the CDC and clinical hospitals, specific antibiotic usage for the enrolled cases were unavailable. This precluded a more in-depth analysis of *Salmonella* antibiotic susceptibility in this study. Due to delays in the feedback of molecular biology test results and the cross-provincial distribution of cold-chain food, the traceability investigation faced difficulties, and the definitive source of exposure was ultimately not identified.

## Conclusions

In summary, this outbreak represents a foodborne event of *Salmonella* Litchfield infections, which was not caused by chef-mediated transmission. Instead, the molecular and epidemiological evidence points to a common-source outbreak linked to a contaminated food item, most likely originating from the Jiangsu-Zhejiang region, although the specific vehicle could not be identified. This study also highlights the value of integrating WGS and mNGS in outbreak investigations. WGS enabled precise characterization of the outbreak clone, as well as its virulence and resistance profiles. mNGS provided an unbiased screening approach that detected *Salmonella* genus-level reads in food samples, supporting the epidemiological link to a contaminated food source; however, the data were insufficient for full genome assembly and strain-level confirmation. By integrating WGS and mNGS, this study not only precisely characterized the genetic features of the outbreak clone, along with its virulence and resistance profiles, but also provided critical evidence for tracing the source of cold-chain food products and promoting cross-regional data sharing. From a food safety traceability perspective, this incident underscores the urgent need to strengthen source tracing of cold-chain foods and to establish interregional data-sharing mechanisms, which would greatly enhance cross-provincial traceability capacity and facilitate early warning and coordinated responses to future foodborne outbreaks.

## Data Availability

The datasets presented in this study can be found in online repositories. The names of the repository/repositories and accession number(s) can be found in the article/Supplementary material.
